# Use of Human Intestinal Enteroids for Recovery of Infectious Human Norovirus from Berries and Lettuce

**DOI:** 10.3390/foods12234286

**Published:** 2023-11-28

**Authors:** Samantha Q. Wales, Michael Kulka, Brianna Keinard, Diana Ngo, Efstathia Papafragkou

**Affiliations:** Office of Applied Research and Safety Assessment, CFSAN FDA, Laurel, MD 20708, USA; michael.kulka@fda.hhs.gov (M.K.); brie.keinard@gmail.com (B.K.); efstathia.papafragkou@fda.hhs.gov (E.P.)

**Keywords:** human norovirus, human intestinal enteroids, virus infectivity, food safety

## Abstract

Norovirus (NoV) is the leading cause of viral foodborne gastroenteritis globally. Currently, the gold standard for detecting NoV in clinical, food, and environmental samples is via molecular-based methods, primarily RT-PCR. Nevertheless, there is a great need for confirmatory assays that can determine the infectivity of viral particles recovered from contaminated matrices. The use of the human intestinal enteroids system (HIEs) has allowed for the expansion of norovirus replication, although it still suffers from limitations of strain preferences and the requirement of high titer stocks for infection. In this study, we wanted to explore the feasibility of using the HIEs to support the replication of NoV that had been recovered from representative food matrices that have been associated with foodborne illness. We first confirmed that HIEs can support the replication of several strains of NoV as measured by RT-qPCR. We subsequently chose two of those strains that reproducibly replicated, GII.4 and GII.6, to evaluate in a TCID_50_ assay and for future experiments. Infectious NoV could be recovered and quantified in the HIEs from lettuce, frozen raspberries, or frozen strawberries seeded with high titers of either of these strains. While many experimental challenges still remain to be overcome, the results of this study represent an important step toward the detection of infectious norovirus from representative produce items.

## 1. Introduction

Norovirus (NoV) is the leading cause of gastroenteritis worldwide, causing over 100,000 hospitalizations, 465,000 emergency department visits, and 2.3 million outpatient visits in the US alone per year [1,2]. Norovirus is a nonenveloped, positive sense, single-stranded RNA virus in the family Caliciviridae that is highly stable in the environment, particularly in cold temperatures [3,4], and can be inactivated at high temperatures and hydrostatic pressure [5,6]. Fresh and frozen produce, mollusks, and ready-to-eat (RTE) foods have been frequently implicated in outbreaks worldwide, with notable large outbreaks caused by soft fruit consumption [7]. One such outbreak in Germany involved 11,000 people who became infected with norovirus-contaminated frozen strawberries [8]. The sewage contamination of water irrigation systems, as well as contact with the stool or vomit from infected food handlers, have frequently been implicated/linked to outbreaks of illness due to contaminated produce [8,9,10,11]. Volunteer studies have shown that very low amounts of viruses are capable of causing illness [12]. Taken together, it is no surprise that detection, prevention, and mitigation strategies are a high priority for food safety experts worldwide. 

Historically, human norovirus has proven difficult to culture [13,14]. Until recently, there was no robust, reproducible cell culture system to support norovirus replication, but since the human intestinal enteroids system (HIEs) was developed, it has been broadly evaluated for the replication of several enteric viruses, including various strains of norovirus [6,15,16,17,18,19,20,21]. While the use of HIEs for the infectivity assessment of human norovirus has proven to be reproducible, it is still hampered by the inability to support all NoV genotypes. Human intestinal enteroids cultures have already been successfully used with noroviruses for efficacy studies, including hand sanitizers, surface disinfection [16,22,23,24], stability in seawater and freshwater [17,20], and for heat inactivation in freshwater clams [25]. However, to our knowledge, there are no published studies on the use of HIEs to identify infectious viruses recovered from fresh or frozen produce.

In this study, we used the human intestinal enteroids system to screen a variety of norovirus genotypes for replication and identify those that demonstrated reproducibly positive virus replication. We selected two different norovirus genotypes (GII.4[P16] and GII.6[P7]) [26] for the establishment of a TCID_50_ assay. Finally, we used the replication-competent strains GII.4[P16] and GII.6[P7] in the enteroids culture system to measure infectious norovirus recovered from a variety of fresh and frozen artificially spiked produce items toward establishing the utility of this culture system for the detection of infectious virus particles recovered from foods. 

## 2. Materials and Methods

### 2.1. Human Intestinal Enteroids

Human jejunal enteroids (J2) were generously provided by Dr. Mary Estes (Baylor College of Medicine, Houston, TX, USA). The enteroids were passaged, and monolayers were prepared on 96-well plates for infection experiments, as reported previously [6,19]. Three-dimensional enteroids were grown in Human IntestiCult Organoid Growth Medium (Components A and B; Stem Cell Technologies, Vancouver, BC, Canada), while monolayers were differentiated in a 1:1 ratio of Component A and CMGF media for 4 days prior to infection. Infections were performed as previously described [6,19] in which 100 µL virus (either stool or vomit suspensions or virus suspensions recovered from produce) was adsorbed to cells for 1 h, washed 3× with CMGF-, and then either frozen immediately (0 h timepoint) or continued incubating for 3–5 days before freezing in a differentiation medium containing 500 µM of the bile acid GCDCA (Sigma-Aldrich, Burlington, MA, USA).

### 2.2. Viruses

Norovirus-positive stools or vomit were obtained either from collaborators (kind gifts from Dr. Suzie Coughlan, UCD National Virus Reference Laboratory, Dublin, Ireland; Dr. Neda Nasheri, Health Canada, Ottawa, Canada; and Julia Wolfe, Orange County Public Health Laboratory, Santa Ana, CA, USA) or from sporadic cases. An amount of 100 µL stool or vomit was mixed with 900 µL PBS, vortexed, sonicated briefly, and then clarified by centrifugation at 25,000× *g* for 5 min at 4 °C. Some stool samples were further filtered onto pre-wet 0.2 µm syringe filters (MillexGP PES membrane, Millipore Sigma, Burlington, MA, USA) and aliquoted. A minimum of 50 µL was extracted for RNA, and RT-qPCR was used to determine virus copy number/µL. For screening and infection experiments, the number of viral genome copies added per well for cell infection is summarized in Table 1. Stool suspensions were diluted as needed (often 1:100 or more) to achieve approximately 100,000 genome copies/well; a minimum dilution of 1:5 of 10% stool was required to avoid general cytotoxicity. Dilutions were made in CMGF containing 500 µM GCDCA. The word “strain” is used in this study for simplicity to denote the genotype determined for a given stool or vomit sample without intent to imply viral purification or adaptation in cell culture. Only stool or vomit suspensions were used to infect cells, with the exception of the GII.4[P16] passaging experiments (Supplementary Materials). In experiments in which the virus was isolated from produce, the term “recovered virus” was used.

### 2.3. TCID_50_ Assays

For determination of the infectious virus titer of the GII.4[P16] and GII.6[P7] fecal filtrates, monolayers of J2 HIEs in 96-well plates were used, with 8 wells for each virus dilution, (2 wells for the 0 hpi time point, and the remaining 6 wells for 5 days post-infection (dpi)) on separate plates. Each assay was performed in triplicate for each virus strain. Viruses were serially diluted at a 1:3 ratio beginning at 1:10 or 1:30 of a 10% fecal suspension, and 6 dilutions were used per virus. For the 0 hpi wells, following a 1.5 h adsorption, plates were washed three times with CMGF media, overlaid with 50 µL CMGF media and stored at −20 °C until RNA extraction of the entire well. For the 5 dpi plate, which was adsorbed and washed in parallel with the 0 h plate, the wells were overlaid with 150 µL differentiation medium containing 500 µM GCDCA, which was replenished with an additional 75 µL medium on day 2 post-infection. Plates that were 5 dpi were frozen and thawed three times, after which 80 µL was removed from each well for RNA extraction to determine total virus RNA copies per well. Individual 5 dpi wells were scored as replication positive if there was a ≥5-fold increase in viral genome copies over the average of genome copies in 0 h wells at the same dilution. Viral genome copy numbers were determined by RT-qPCR analysis as described in Section 2.6. The infectious dose 50% (ID_50_) genomic copies and tissue culture infectious dose 50% (TCID_50_) for infectivity of these two strains was determined by applying the Reed–Muench method as previously reported and described for human norovirus replication in HIEs [16]. The application of a modified Karber method, as discussed in [27], yielded similar values for TCID_50_ and was used to determine 95% confidence intervals for the respective genotypes.

### 2.4. Virus Recovery from Foods

Virus extraction was completed as described in US FDA BAM chapter 26 [28] without the inclusion of process control virus and with the modifications described below. Briefly, frozen strawberries, frozen raspberries, or fresh lettuce were purchased from a local supermarket, thawed (if frozen), and, in the case of strawberries, cut into smaller pieces. An amount of 50 g of produce was weighed into a filtered Whirl-Pak plastic bag and inoculated with 100 µL of 10% stool suspension in PBS from either norovirus GII.4[P16] or GII.6[P7] (approximately 5 × 10^7^ genome copies per virus). After 1 h incubation at room temperature, food was stored overnight at 4 °C. The following day, the virus was eluted and concentrated with ultracentrifugation, as described in BAM chapter 26. The concentrated pellet was resuspended in 600 µL of enteroids infection media (CMGF—with 500 µM GCDCA with 100 units Pen/mL/100 µg/mL Strep added; a volume of 600 µL was chosen to accommodate the addition of 100 µL to 4 wells for the 0 h and 3 dpi plus enough remaining to extract RNA to calculate genome copies added per well), sonicated for 5 min, and spun at 13,800 rpm for 5 min at 4 °C in a microcentrifuge to clarify the virus particles from accumulated solids. In some experiments, a portion of the recovered virus was further diluted as indicated using CMGF with GCDCA and Pen/Strep. The virus extracts were stored at −80 °C prior to use in culture or RNA isolation but typically were used within 1–2 weeks after recovery. 

### 2.5. RNA Extraction and Purification

RNA extraction of infected cells and viral inputs (including the virus recovered from berries and lettuce) was performed using Trizol™ (Life Technologies, Carlsbad, CA, USA) to lyse the samples, followed by the use of the Direct-zol RNA extraction kit, following the manufacturer’s instructions (Zymo Research, Irvine, CA, USA). RNA recovered from berries was purified using the PCR Inhibitor Removal Kit (Zymo Research), according to the manufacturer’s instructions. 

### 2.6. Detection by Real-Time RT-qPCR

Quantitative reverse-transcription PCR (RT-qPCR) on 5 µL RNA (input and cell) extracts was performed as described previously [19]. A full-length GII.4 RNA transcript was used for the standard curve [29], and all PCR reactions were run on an ABI 7500 FAST machine. Standard curves were linear (r^2^ ≥ 0.98) over the concentration range of 1.7 × 10^5^ to 17 genomic copies/5 µL RNA per reaction with slopes indicating reaction efficiencies from 91 to 98%; corresponding Ct values ranged from 22.57 to 39.03, respectively. Deviation from linearity (Ct vs. genome copy number per reaction) and decreased detection (signal) frequency of <100% (signal) occurred at concentrations of <17 copies/5 µL RNA per reaction. In order to (i) provide a minimum value for “quantification” on the nonlinear portion of the curve, (ii) acknowledge that an “undetermined” signal may represent either an absence of an RNA target or the presence of a target at a concentration that may give <100% detection frequency, and (iii) limit overestimation of fold increase for samples containing low copy numbers, we assigned a value of 8 genome copies/5 µL RNA per assay to “undetermined” signals from 0 hpi where 3 to 5 dpi yielded a Ct value. The 8 genome copies/5 µL RNA thus convert to 128 genome copies in an 80 µL volume of extract. 

### 2.7. Statistical Analysis

Analysis of variance (ANOVA) and linear regression analyses were used to evaluate standard curves and assay performance using GraphPad Prism (version 9.5.1).

## 3. Results

### 3.1. Screening of NoV Fecal Filtrate Replication with J2 HIEs

We established a successful culture of the HIEs in our laboratory and set about screening the 40 fecal filtrates of norovirus that we acquired (Table 1). Our threshold for defining whether a sample is replication positive was based on a minimum 5-fold increase in genome copies from t0 dpi (1 h post adsorption) to 3–5 dpi as previously described [18]. Of the 40 filtrates tested, 12 were replication-positive, and all except 1 GI were GII strains. The majority of the GII replication-positive genotypes were GII.4 (7 samples), with three GII.6, one GII.1, and one GI sample that also showed a 5-fold increase or more. The GI strain was not genotyped. In the negative-replicating cohort, there were three GII.4, six GII.6 (one of which was vomit), one GII.1, one GII.7 (vomit), one GII.17, one GI.6, five GI.3, two GII samples not typed, and eight GI samples not typed. We had one paired set of GII.6[P7] stool and vomit, in which the NoV from stool [26] replicated, but not that from the vomit. We also had a second paired set (GII.1) that was acquired from an adult and child within the same household [30]. In this set, the virus from the child’s sample did not replicate despite having a very high titer (average of 7.20 × 10^5^ genome copies added per well at a 1:200 dilution of the 10% filtrate). The virus from the adult’s sample did replicate, although at lower levels of efficiency than for those typically seen with some of the GII.4 strains (range of 11–a 200-fold increase from t0). During the screening process, we observed that one strain of GII.4[P16] occasionally caused a cytopathic effect (observable by discrete, dark/dense cells that have become unattached from the monolayers in a non-uniform manner; see Supplemental Figure S2 as an example) and exhibited high RNA copy titers after three days of replication. We chose to further examine the replication phenotype of this strain (see Supplemental Figures S1 and S3) as well as for the GII.6[P7] strain [26] from stool (both indicated in bold in Table 1), as we had relatively large volumes of samples that had also demonstrated consistent replication. 

### 3.2. Characterization of Culture Conditions for GII.4[P16] and GII.6[P7] Genotypes

**Figure 1:**
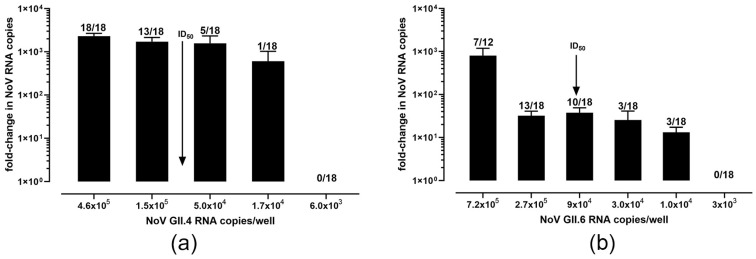
Calculated TCID_50_ of two in-house strains of human norovirus. (**a**) ID_50_ (RNA copies) ≈ 8.1 × 10^4^ copies/well (at 1 TCID_50_) for GII.4[P16] strain. (**b**) ID_50_ (RNA copies) ≈ 9.7 × 10^4^ copies/well (at 1 TCID_50_) for GII.6[P7] strain.

Our first goal was to determine the ID_50_ and TCID_50_ of these two genotypes whereby all three replicates (from three separate infections) were averaged to obtain the data for the GII.4[P16] (Figure 1a) and GII.6[P7] (Figure 1b) strains. The ID_50_ of the GII.4[P16] was determined as 8.1 × 10^4^ genome copies (at 1 TCID_50_); e.g., for a given set of wells, 8.1 × 10^4^ gc per well are required to successfully infect (a five-fold increase over t0) half of the wells. A 570 TCID_50_ (95% CI: 125, 2512)/1 µL of 10% stool suspension was determined for GII.4[P16. The ID_50_ for GII.6[P7] was 8.8 × 10^4^ genome copies (at 1 TCID_50_) and 81 TCID_50_ (95% CI; 10, 676)/1 µL of 10% suspension. 

### 3.3. Infection of J2 HIEs with Virus Recovered from Frozen Raspberries

Having established that both the GII.4[P16] and GII.6[P7] viruses consistently replicate in the J2 HIEs, we wanted to determine the feasibility of using this cell culture system to measure infectious virus recovered from different produce matrices using the FDA BAM protocol for produce as described in chapter 26 [28] with the modifications described in the Materials and Methods section. We began our investigation using frozen raspberries, as they have been implicated in numerous foodborne norovirus outbreaks. Preliminary trials revealed that the chloroform step used to remove extraneous PCR inhibitors yielded viral extracts that were toxic to the enteroids’ monolayers and resulted in massive cell death, so this step was omitted in future experiments (Table 2). Additionally, rather than resuspending the viral pellet after ultracentrifugation in PBS, we used CMGF containing antibiotics and the bile acid GCDCA (500 µM) in order to prepare the samples for direct application in enteroid cell culture infections. Frozen raspberries were spiked with 10% suspensions of either GII.4[P16] or GII.6[P7] (approximately 5 × 10^7^ genome copies per sample) on three separate occasions, generating enough viral samples to perform infections on four batches of cells (one batch was 4 wells of a 96-well plate; 2 wells per time point per sample) of the recovered virus. Cytotoxicity was never observed in control wells containing samples derived from unspiked produce, and a positive control (derived from stool resuspension) was included in each experiment. After extraction from raspberries, only 3 out of 8 virus inputs for GII.4[P16] and 1 of 6 for GII.6[P7] could be detected by RT-qPCR prior to their addition to cells, even after the use of a PCR inhibitor removal kit on all the sample inputs, followed by a 1:10 dilution of the RNA (Table 3). Despite this treatment, we believe the absence of an amplification signal for the majority of these “negative input” samples was due to the presence of PCR inhibitors because four or six of these samples yielded readily detectable signals at 0 hpi for GII.6[P7] and GII.4[P16] viruses, respectively. Replication (defined in this study as a >5-fold increase in PCR signal from 0 h to 72 h pi) was confirmed in all but one sample of virus recovered from raspberries, with fold increases ranging from 14 to 163,000. In one extracted sample, the virus (GII.6[P7]) was undetectable in the input, as well as at 0 and 72 h post-infection, suggesting an absence of recovered virus or at levels below quantification. For the GII.4[P16] recovered virus, we observed a cytopathic effect by 3 dpi in six out of eight infections performed (Supplementary Figure S4). 

### 3.4. Infection of J2 HIEs with Virus Recovered from Frozen Strawberries

In order to investigate the performance of the virus isolation method in the context of infectious virus recovery and whether there is a significant difference in infection rates of virus recovered from berries of a different type, we performed another set of experiments using frozen strawberries as they are a popular fruit that is commonly consumed raw. The experiments with strawberries were performed in duplicate, generating enough samples for three separate infection experiments (Table 3). In one set of experiments, we diluted a portion of the recovered virus (GII.6[P7] or GII.4[P16]) at 1:3, 1:10, and 1:20 to begin exploring the minimum infectious dose. The GII.6[P7] strain was undetectable (Udt) in 6 of the 11 input samples with variable estimates of the recovered virus despite a 20-fold dilution range suggesting the presence of PCR inhibitors carried over from the strawberry extraction. While all of the GII.4[P16] inputs (12/12) were detectable, quantification revealed similar variability in recovery. On the other hand, the undetected 3 of 12 0 hpi time points for the GII.4[P16] strain and 3/11 GII.6[P7] strain are likely due to either unrecovered virus or recovered at levels below assay detection limits. In contrast to the GII.6[P7] virus recovered from raspberries (positive in 5 of 6), only 3/11 samples tested positive for replication when recovered from strawberries. However, the GII.4[P16] strain retained its consistency for replication with the majority (10/12) of samples positive at 3 dpi. It should be noted that we did not observe a cytopathic effect in any of these cells. The fold increase in genome copies from 0 h to 3 dpi varied between replicate samples but overall displayed similar levels of replication/variation compared to virus infection without extraction from the food matrix. 

### 3.5. Infection of J2 HIEs with Virus Recovered from Fresh Lettuce

We chose to use fresh lettuce as the third matrix to investigate the recovery of infectious norovirus from foods (Table 3). In addition to having a different composition than berries, it is a representative of produce consumed raw and has also been implicated in a number of foodborne outbreaks [31]. Similar to the experiments with berries, fresh romaine lettuce was spiked with either of the two strains on three separate occasions, allowing for three separate infections with duplicate samples. Some samples were diluted 1:10 after recovery prior to infection. All inputs and 0 hpi time points for both virus strains were detectable by RT-qPCR, although four were below our estimated limit of detection. There appears to be little correlation between viruses present/bound to cells at 0 hpi and positivity for replication. Similar to the results obtained from strawberries and raspberries, the GII.4[P16] strain consistently replicated in the enteroids when extracted from the majority of lettuce samples (9 of 11), while GII.6[P7] only replicated in 2 of 12 samples following extraction from lettuce.

## 4. Discussion

Detection methods for human noroviruses have traditionally relied primarily on RT-PCR [32,33,34], which is capable of detecting low quantities of viral genome but inherently cannot distinguish between infectious and non-infectious virus. As such, the importance of infectivity assays, developed to complement results from molecular-based testing, is now being fully recognized to assess the comprehensive public health risk [9,12,33,35,36]. Identifying infectious human norovirus originating from naturally contaminated foods remains a challenge in food safety as viral contamination levels are typically very low and often not homogeneously dispersed [8]. In this study, we aimed to first establish and validate the HIE culture system with a variety of strains and subsequently determine and present results on the utilization of this system to recover infectious norovirus from representative foods.

We first established the HIE culture and maintenance conditions in our laboratory and screened our existing norovirus bank of strains to identify those that would grow in culture. Via testing forty different samples, we found the majority that could successfully replicate belonged mostly to genogroup II and were more specifically GII.4 strains. It is interesting to note that some strains that were tested were of the same genotype (see Table 1, GII.4[P16] strains) yet differed in their ability to grow in the HIEs. It has been previously demonstrated, and we show here that high numbers of genomic copies (as determined by RT-qPCR) are required for successful infection of the HIEs [6,18], although this could be due to high levels of inactivated/non-infectious or defective virus particles, which can still be measured by PCR [16]. Indeed, we observed that high copy numbers alone did not determine success in replication, as strains belonging to both GI and GII genogroups have been shown to replicate in this system [6,16,18,19]. We postulate that perhaps differences in stool compositions within samples can greatly affect the efficiency of norovirus replication in HIEs, and we are currently seeking experimental designs to test this hypothesis. An alternative reason for the limitations in norovirus growth could be due to the lack of the microbiome in the HIE system, as others have indicated the importance of some bacterial strains for norovirus growth [37,38]. When we tested two samples obtained from vomit (GII.7 and GII.6[P7], 1.5 × 10^5^ and 5.4 × 10^4^ genome copies added/well, respectively), neither of these replicated, although input titers were not unusually low. A recent publication from Hagbom et al. [39] showed that vomit samples belonging to the GII.4 genotype and with high titers (Ct values of <26, 8.9 × 10^6^ to 1.6 × 10^10^ GEq/mL) could successfully replicate in HIE cultures. There has been considerable evidence on how vomit carries infectious virus particles that can help spread the disease [12,40], so it is unclear why these samples were negative. Alternatively, the lack of replication from our vomit-derived virus may be a function of their genotypes (GII.7 and GII.6[P7]). Overall, despite the limitations in identifying a large number of different strains that consistently and robustly replicated (i.e., high titers based on RT-qPCR), we did find two that came close to these requirements, which we further characterized prior to using them for spiking experiments. 

The GII.4[P16] strain (referred to as GII.4 (011617) in [19]) replicated consistently and robustly, following the trend of many GII.4 strains tested in HIEs by our and other laboratories. Interestingly, this is the first norovirus strain for which we observed a cytopathic effect (see Figure S2), which we previously had not observed, although others have seen cpe in HIEs infected with GII.4 [6]. This was typically only observed in HIEs at lower passage numbers (*p* < 20), highlighting the importance of the overall health of HIE cultures for successful norovirus replication. Indeed, this system is extremely sensitive to external environmental factors, such as medium composition, as we and others have recently observed, to affect the ability of noroviruses to replicate (unpublished observations). Given that some laboratories use commercial media, while others use “in-house” media dependent on the use of conditioned media for several components (Wnt, R-spondin, and Noggin) derived from either one cell line (L-WRN, ATCC) or three cell lines [6] to compose it, it is no surprise that different levels of success can be achieved with replication of norovirus by different investigators in this system. 

We determined the TCID_50_ of two genetically distinct genotypes (GII.4[P16] and GII.6[P7]) using a slightly more statistically robust method than previously published [16] by diluting viral inputs 1:3 rather than 1:10 and infecting six wells per dilution versus three. We adopted the use of RT-qPCR as the endpoint determination for viral tissue culture infectious doses, using a five-fold increase in genome copies as cut-off, the parameters that we and others have used for determining positive norovirus replication [6,16], based on the work of other laboratories using quantitative PCR as endpoint determination [41,42]. Our ID_50_ values for both strains tested were higher than was previously reported (1 × 10^3^ [6,16]); however, comparisons are limited when utilizing different stool samples despite the similarity in genotypes. Indeed, given (i) the presence of an unknown and potentially large number of defective particles in the stool that may vary with collection time post-infection, (ii) the mixed cell population of the differentiated monolayers having varying susceptibility to NoV infection, and (iii) the variance in general “culture health” from passage to passage, the routine application of a TCID_50_ assay toward determination of infectious viral titers will require further investigation. We believe it is only feasibly useful for fecal samples used experimentally rather than for samples that may be recovered from contaminated food sources, given the relative paucity of viral particles that can be recovered from these samples and the relatively large amounts required to perform a rigorous TCID_50_ assay such as ours. However, it would be interesting to determine the TCID_50_ values of stools collected from a patient throughout the course of illness to examine how the infectious dose/particle number might change with time.

We also further characterized the kinetics of GII.4[P16] infection in the supernatant and cellular fractions from 1 to 5 dpi. Similar to what has been reported [6,16], we observed a peak of virus copies in the supernatant at days 2–3 post-infection (Figure S1A), with additional accumulation of viral genome copies evident in samples collected 5 dpi (Figure S1B). Because we sometimes observed a cytopathic effect in the GII.4[P16], we wanted to determine if this robustly replicating virus could be passaged further than the previously tested GII.4 genotype, which diminished after passage 4 in the J2 line, although higher passages of NoV have been obtained in enteroids derived from iPSCs [6,43]. We decided to pool wells that exhibited cytopathic effects to see if we could select for a cytopathic/culture-adapted strain (Figure S3A). As shown in Figure S3B, we observed successful replication (>5-fold increase in virus) in two of three cytopathic replicates up to passage 5, and in one replicate, we were able to see productive infection through passage 7, although we lost the cytopathicity after passage 4 in all replicates. 

Standardized, validated methods that are currently used for virus recovery and detection in foods [28,44] are both PCR-based; however, the virus extraction part can recover virus particles that, in principle, can be used for infectivity assays. Measuring infectious virus from foods or other naturally contaminated samples, which requires the use of additional filtration and purification steps to remove residual food components and/or bacteria/fungi that can be toxic to cells, has frequently been cited to be challenging even for more established cell lines [45,46]. PCR inhibition in food samples has been widely witnessed and especially in berries, inhibition can be considerable and variable even between different fruit lots [8,35,47]. Commonly, a chloroform extraction step is performed to remove copurified PCR inhibitors from viral concentrates. Since we had to eliminate this step due to lingering cell toxicity, we ended up with several samples that were not detectable by RT-qPCR. For much of the work with the frozen berries, we were not able to directly measure how much virus was used to infect the J2 HIE monolayers as the inoculum was undetectable, either because it was below the limit of the RT-qPCR assay or due to the presence of PCR inhibitors [35,48,49,50]. In several cases where there was still some RNA left recovered from foods, we purified and re-tested it with the same RT-qPCR assay; indeed, even 1:10–1:20 dilution of the RNA was insufficient to allow detection, although it cannot be ruled out that dilution caused the genome copies present to be reduced below the limit of detection. Surprisingly, in these studies, there was significant replication in some samples, even when the inoculum could not be quantified, which, at this point, we attribute to PCR inhibition. There were also a few instances where the 0 hpi time point (post-virus adsorption) was undetectable, but robust replication occurred. In these instances, we assumed the levels of virus present were below the limit of detection (which we estimated as being <128 copies/well); this was unlike what was witnessed with the stool filtrates, where replication was feasible when input RNA copies were, depending on the genotype, at least 100,000 copies [16,21,39] and the 0 hpi time points were always detectable and typically in at least the 1 × 10^3^ range. 

It has been suggested that different NoV genotypes may perform differently in terms of environmental persistence, inactivation patterns, and binding to putative receptors such as histo-blood group antigens, fucose, etc. [51,52]. In a more recent study using the HIE system, GII.3 and GII.4 showed similar stability when stored in seawater at 12 °C for up to five weeks [17]. In our study, we observed that GII.4[P16] showed more consistent levels of replication than GII.6[P7] after being recovered from artificially seeded foods, but this may be partially attributed to the preferential susceptibility of the J2 HIE monolayers for one genotype as previously reported [39], or due to the relatively reduced ID_50_/TCID_50_ values for this particular genotype. For the experiments conducted with lettuce, we were able to calculate recoveries that ranged from 0.3 to 12% for GII.4[P16] and 0.6–5% for GII.6[P7], similarly variable to other studies with HIEs [21] or studies using Nov surrogates [53,54]. Overall, we observed considerable variability among replicates of the same virus and matrix in the fold change in viral genomes. As we sought to evaluate our BAM-based protocol for both recovery of infectious virus and viral nucleic acid, the occasional lack of reproducibility between measured fold increase in virus RNA titers may be due to the method’s not being optimized and virus particles being lost or rendered incapable of replication in HIE cells. The recovery efficiency of this method, however, cannot be directly compared to other studies as the virus used, the quantity of product tested, as well as the methodology used could all contribute to such variability. 

Our study had several limitations. We used a high-titered virus as the inoculum for the raspberry, strawberry, and lettuce seeding studies as a proof-of-concept study and an inoculum level that may be closer to what has been often found in naturally contaminated foods (typically closer to 10^2^ to 10^3^ genome copies per gr [47,48]. Indeed, the current requirement of the HIEs for high-titered viruses to ensure successful replication is a large hindrance that must be overcome to ensure success with actual NoV-contaminated outbreak-associated foods. Future studies should be aimed at increasing the sensitivity threshold of the assay, as well as expanding the susceptibility of the HIEs to more NoV genotypes. Overall, the HIE system shows promise for use in the detection of infectious NoV GII virus particles originating from multiple fresh or frozen contaminated produce types despite the hurdles that must be overcome to improve the assay.

## Figures and Tables

**Table 1:** Summary of human norovirus strains tested in J2 HIEs. RNA cp refers to estimated genome copies added/well at the time of infection. Fold-inc was determined by dividing the total copy numbers per well at 3 dpi by the total copy numbers per well at 0 h pi. * Indicates stools obtained from sporadic cases. NVRL samples provided by Dr. Suzie Coughlan, NVRL, UCD Dublin, Ireland; + samples provided by Dr. Neda Nasheri, Health Canada, Ottawa, CN; ^ samples provided by Julia Wolfe, Orange County Public Health Laboratory, Santa Ana, CA. The two NoV strains in bold were used in further studies.

Positive (≥5-Fold Increase)		Negative (≤5-Fold Increase)				
Genotype:	RNA cp:	Fold-inc:	GII Genotype:	RNA cp:	GI Genotype:	RNA cp:
GII.4 Sydney (BCM)	3.00 × 10^5^	2.9 × 10^3^	GII.4 (2014) *	2.90 × 10^5^	GI.6[P11] *	1.02 × 10^6^
GII.4[P16] (011617) *	1.98 × 10^5^	1.4 × 10^4^	GII.4[P16] (1043 NVRL)	9.50 × 10^4^	GI.3[P3] (#2) ^	3.83 × 10^7^
GII.4[P16] (1036 NVRL)	4.00 × 10^5^	1.4 × 10^3^	GII.4 NO/Sydney (16-78)+	2.60 × 10^6^	GI.3[P3] (#3) ^	6.36 × 10^6^
GII.4[P16] (1041 NVRL)	4.70 × 10^4^	2.1 × 10^3^	GII.6 (15-65)+	3.10 × 10^4^	GI.3[P3] (461-1) ^	5.06 × 10^3^
GII.4[P16] (1046 NVRL)	8.00 × 10^5^	5.0 × 10^0^	GII.6[P7] (2014 vomit) *	5.40 × 10^4^	GI.3[P3] (461-2) ^	6.38 × 10^5^
GII.4 Sydney (13-39)+	5.60 × 10^5^	2.4 × 10^3^	GII.6[P7] EP3 *	1.80 × 10^3^	GI.3[P3] (461-3) ^	2.16 × 10^5^
GII.4 Sydney (15-59)+	2.60 × 10^5^	1.6 × 10^2^	GII.6[P7] EP4 *	1.40 × 10^4^	GI *	3.80 × 10^6^
GII.6[P7] (2014 stool) *	1.60 × 10^5^	4.2 × 10^2^	GII.6[P7] EP5 *	3.20 × 10^5^	GI (1048 NVRL)	3.64 × 10^5^
GII.6 (14-55)+	1.70 × 10^5^	1.9 × 10^1^	GII.6[P7] EP6 *	6.60 × 10^5^	GI (1049 NVRL)	3.32 × 10^4^
GII.6 *	7.64 × 10^4^	8.0 × 10^0^	GII.1 SD (2016) *	7.20 × 10^5^	GI (1052 NVRL)	5.75 × 10^2^
GII.1 JW (2016) *	4.10 × 10^5^	8.4 × 10^1^	GII.7 (vomit) *	1.50 × 10^5^	GI (1054 NVRL)	9.62 × 10^6^
GI (1257 NVRL)	2.12 × 10^6^	8.0 × 10^0^	GII.17 (118-2)^	9.92 × 10^4^	GI (1057 NVRL)	1.07 × 10^5^
			GII (1038 NVRL)	2.65 × 10^3^	GI (1249 NVRL)	1.39 × 10^5^
			GII (1040 NVRL)	1.42 × 10^5^	GI (1252 NVRL)	8.98 × 10^3^

**Table 2:** Protocol for modified BAM method of recovery and infection of virus particles from frozen fruit or fresh lettuce for cell culture.

Weigh 50 g of food and inoculate with 100 µL of 10% stool suspension (~5 × 10^7^ gc)
Let air dry for 1 h at RT
Elute virus with 0.1 M Tris-HCl-0.05 M glycine-1% beef extract buffer, pH 9.2 with Polyvinylpyrrolidone (PVP), and pectinase on a shaking platform (30 min at RT)
Clarify eluate with centrifugation at 10,000× *g* at 4 °C
Concentrate the virus by ultracentrifugation at 170,000× *g* for 1 h at 4 °C
Resuspend pellet in 600 µL media; sonicate and clarify particles at 4500 rpm/5 min
Infect enteroids (100 µL/well; 1 h adsorption; 3 washes) and collect samples at times 0 and 3 dpi
Extract RNA from cells and supernatants with TrizolQuantify virus recovery from foods and replication by RT-qPCR with in-house full-length transcripts

**Table 3:** Infection of J2 HIEs with GII.6[P7] or GII.4[P16] virus recovered from frozen raspberries, frozen strawberries, or fresh lettuce. Results are presented as the average genome copy number per infected well at 0 and 72 hpi and the total viral copy number as input for well infection according to the dilution of input (genome copies/well; Udt is undetected). The +/− indicates virus replication as determined by a >5-fold increase from 0 to 72 hpi. * Indicate samples where either there was a signal, but it was lower than the detection limit that we deemed as 128 copies/well, or there was no signal (Udt), but 128 was used to avoid overestimating the fold increase in replication.

Produce Matrix	Sample	Dilution	Input	0 hpi	72 hpi	Fold Increase (Replication +/−)
Frozen Raspberries	GII.6[P7]	None	Udt	1.3 × 10^2^ *	4.2 × 10^4^	3.2 × 10^2^ (+)
			Udt	Udt	Udt	0 (−)
			Udt	3.2 × 10^2^	6.7 × 10^4^	2.1 × 10^2^ (+)
			Udt	3.9 × 10^2^	4.2 × 10^6^	1.1 × 10^4^ (+)
			Udt	5.9 × 10^2^	1.9 × 10^7^	3.3 × 10^4^ (+)
			3.4 × 10^2^	1.2 × 10^3^	1.4 × 10^7^	1.2 × 10^4^ (+)
	GII.4[P16]		Udt	1.3 × 10^2^ *	2.6 × 10^5^	2.0 × 10^3^ (+)
			Udt	1.3 × 10^2^ *	2.0 × 10^5^	1.5 × 10^3^ (+)
			Udt	6.1 × 10^2^	1.0 × 10^8^	1.6 × 10^5^ (+)
			1.2 × 10^3^	7.9 × 10^2^	3.7 × 10^4^	4.6 × 10^1^ (+)
			Udt	2.7 × 10^3^	1.1 × 10^8^	3.9 × 10^4^ (+)
			Udt	4.4 × 10^3^	1.3 × 10^8^	3.1 × 10^4^ (+)
			1.6 × 10^5^	6.4 × 10^3^	6.0 × 10^7^	9.4 × 10^3^ (+)
			2.4 × 10^5^	7.0 × 10^3^	1.0 × 10^5^	1.4 × 10^1^ (+)
Frozen Strawberries	GII.6[P7]	none	Udt	4.0 × 10^2^	2.6 × 10^7^	6.5 × 10^4^ (+)
			Udt	1.5 × 10^3^	1.4 × 10^4^	9.5 × 10^0^ (+)
			2.5 × 10^2^	3.7 × 10^2^	2.0 × 10^6^	5.4 × 10^3^ (+)
			7.1 × 10^2^	Udt	Udt	0 (−)
		1:3	1.8 × 10^3^	2.8 × 10^2^	Udt	0 (−)
		1:10	Udt	Udt	Udt	0 (−)
			Udt	1.3 × 10^2^ *	Udt	0 (−)
			Udt	2.5 × 10^2^	5.1 × 10^2^	2.0 × 10^0^ (−)
			5.1 × 10^1^	Udt	Udt	0 (−)
		1:20	Udt	2.0 × 10^2^	4.0 × 10^2^	2.0 × 10^0^ (−)
			4.7 × 10^3^	8.8 × 10^2^	Udt	0 (−)
	GII.4[P16]	none	7.4 × 10^2^	1.3 × 10^2^ *	5.9 × 10^7^	4.6 × 10^5^ (+)
			1.1 × 10^3^	1.3 × 10^2^ *	7.8 × 10^6^	6.1 × 10^4^ (+)
			5.4 × 10^3^	6.8 × 10^2^	1.0 × 10^7^	1.5 × 10^4^ (+)
			6.9 × 10^4^	4.1 × 10^2^	5.6 × 10^5^	1.4 × 10^3^ (+)
		1:3	1.0 × 10^2^	3.2 × 10^2^	2.7 × 10^5^	8.4 × 10^2^ (+)
			4.0 × 10^3^	2.8 × 10^2^	1.6 × 10^7^	5.7 × 10^4^ (+)
		1:10	6.3 × 10^1^	1.3 × 10^2^ *	1.7 × 10^7^	1.3 × 10^5^ (+)
			1.8 × 10^2^	1.3 × 10^2^ *	1.4 × 10^6^	1.1 × 10^4^ (+)
			2.3 × 10^2^	1.3 × 10^2^ *	1.1 × 10^2^	9.0 × 10^−1^ (−)
			3.5 × 10^2^	Udt	Udt	0 (−)
		1:20	4.8 × 10^1^	1.5 × 10^2^	1.1 × 10^1^	7.1 × 10^−2^ (−)
			4.0 × 10^2^	1.4 × 10^2^	1.9 × 10^5^	1.4 × 10^3^ (+)
Fresh Lettuce	GII.6[P7]	None	2.6 × 10^4^	1.9 × 10^2^	1.5 × 10^2^	7.9 × 10^−1^ (−)
			3.3 × 10^4^	6.1 × 10^2^	5.6 × 10^2^	9.3 × 10^−1^ (−)
			5.6 × 10^4^	3.1 × 10^2^	Udt	0 (−)
			9.7 × 10^4^	9.6 × 10^2^	3.4 × 10^3^	3.5 × 10^0^ (−)
			2.0 × 10^5^	1.4 × 10^3^	6.5 × 10^2^	4.7 × 10^−1^ (−)
			3.7 × 10^5^	5.7 × 10^2^	1.3 × 10^2^	2.3 × 10^−1^ (−)
			5.1 × 10^5^	3.0 × 10^2^	6.4 × 10^3^	2.1 × 10^1^ (+)
		1:10	2.0 × 10^4^	2.2 × 10^2^	Udt	0 (−)
			3.1 × 10^4^	2.9 × 10^2^	Udt	0 (−)
			4.0 × 10^4^	4.1 × 10^2^	1.5 × 10^2^	3.6 × 10^−1^ (−)
			5.0 × 10^4^	2.7 × 10^2^	4.2 × 10^2^	1.5 × 10^0^ (−)
	GII.4[P16]	None	6.1 × 10^4^	6.3 × 10^2^	4.4 × 10^5^	7.0 × 10^2^ (+)
			7.2 × 10^4^	2.0 × 10^2^	2.3 × 10^5^	1.2 × 10^3^ (+)
			8.0 × 10^4^	2.7 × 10^2^	7.4 × 10^3^	2.8 × 10^1^ (+)
			1.3 × 10^5^	1.3 × 10^2^ *	6.6 × 10^4^	5.2 × 10^2^ (+)
			1.6 × 10^5^	5.1 × 10^2^	7.8 × 10^5^	1.5 × 10^3^ (+)
			3.1 × 10^5^	3.6 × 10^2^	Udt	0 (−)
			3.4 × 10^5^	4.0 × 10^3^	1.1 × 10^6^	2.7 × 10^2^(+)
			1.2 × 10^6^	9.0 × 10^2^	1.8 × 10^7^	1.9 × 10^4^ (+)
		1:10	1.4 × 10^4^	1.3 × 10^2^ *	1.3 × 10^2^	1.0 × 10^0^ (−)
			1.6 × 10^4^	1.3 × 10^2^ *	Udt	0 (−)
			2.2 × 10^4^	1.3 × 10^2^ *	9.4 × 10^4^	7.3 × 10^2^ (+)
			1.2 × 10^5^	1.3 × 10^2^	5.2 × 10^5^	3.9 × 10^3^ (+)

## Data Availability

The data presented in this study are available upon request from the corresponding author.

## Supplementary Materials

The following supporting information can be downloaded at https://www.mdpi.com/article/10.3390/foods12234286/s1. Figure S1: GII.4[P16] stool filtrate replication in cells and supernatants; Figure S2: GII.4[P16] NoV-infected cells sometimes cause cytopathic effect in J2 HIEs; Figure S3: Passage of GII.4[P16] virus in J2 HIEs; Figure S4: Cytopathic effect in J2 enteroids infected with GII.4[P16] but not GII.6[P7] norovirus isolated from frozen raspberries.
