# Supplementary material for: Use of Human Intestinal Enteroids for Recovery of Infectious Human Norovirus from Berries and Lettuce
Source: Foods. 2023 Nov 28;12(23):4286. doi: 10.3390/foods12234286 (PMC10706681; doi:10.3390/foods12234286)
Supplement: Supplementary file 1 [file foods-12-04286-s001.zip › foods-2665551-supplementary.pdf]

Supplementary Materials

The GII.4[P16] strain was used to further characterize norovirus replication in the HIE system, because it demonstrated more consistent and efficient replication than the GII.6[P7] strain (Figure 1). We set up duplicate wells in which the supernatant was collected and replenished daily, and duplicate wells in which both the cells and companion supernatants were collected each day for five days, in addition to one well that was collected immediately after virus adsorption (0h). All wells were washed 3x with CMGF- 1h post virus adsorption prior to the start of the experiment. When supernatants were collected either for replenishment or for separation from the cell fraction, cell monolayers were not additionally washed. In the replenished supernatants, we saw a peak of viral genome copies on day 3 in one well, and at day 2 in the other well (Fig. S1A). In both wells, after the peak there was a gradual decline in genome copies/well until the final day. For the cells and supernatants collected daily, there was a general increase in virus copies each day, which is to be expected as viral genome copies accumulate over time, though one replicate had a low total copy numbers on day 3 (Fig. S1B). We observed much greater copy numbers in the cell fraction, but because we did not rinse the monolayers immediately after removing the supernatant for analysis, it is conceivable that putative virus particles (resulting in accumulated genome copies) associated with cell surfaces could have contributed to the difference between the fractions. It is interesting to note that in both replicates, the genome copies in the supernatant fraction were noticeably increased at day 5 post-infection, again reflecting the accumulation of virus within the supernatant over time, like that observed when supernatant is sampled daily (figure S1A).

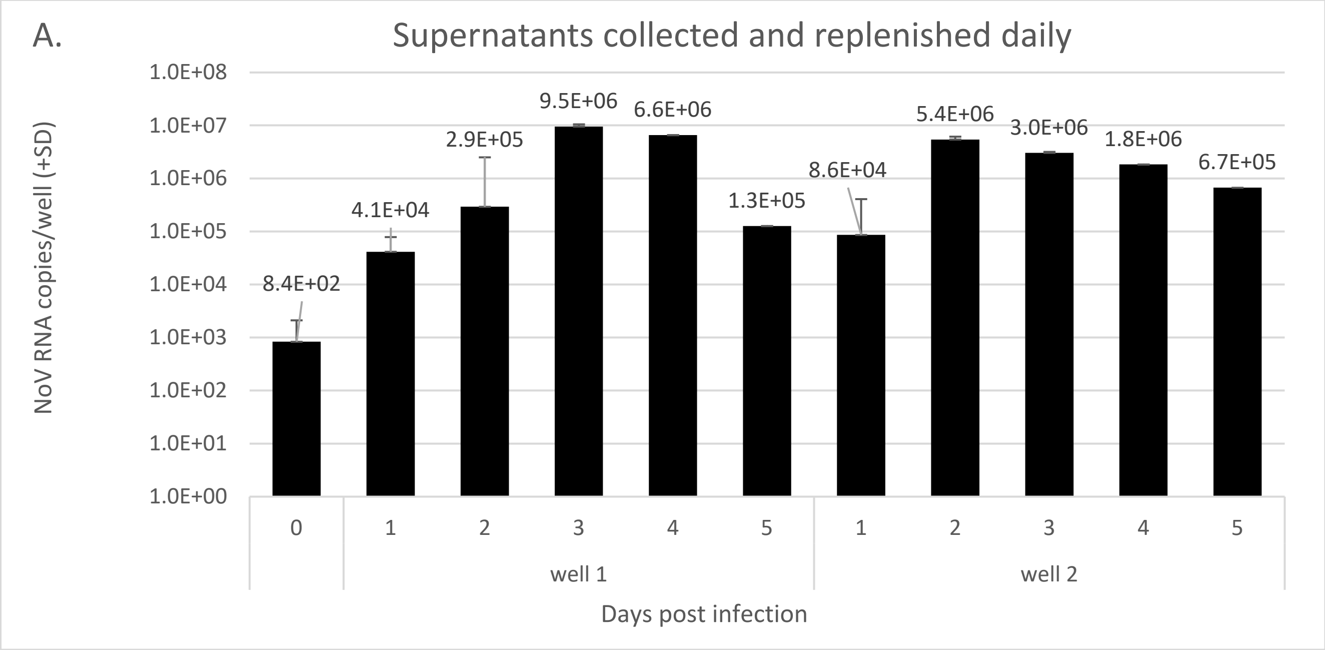

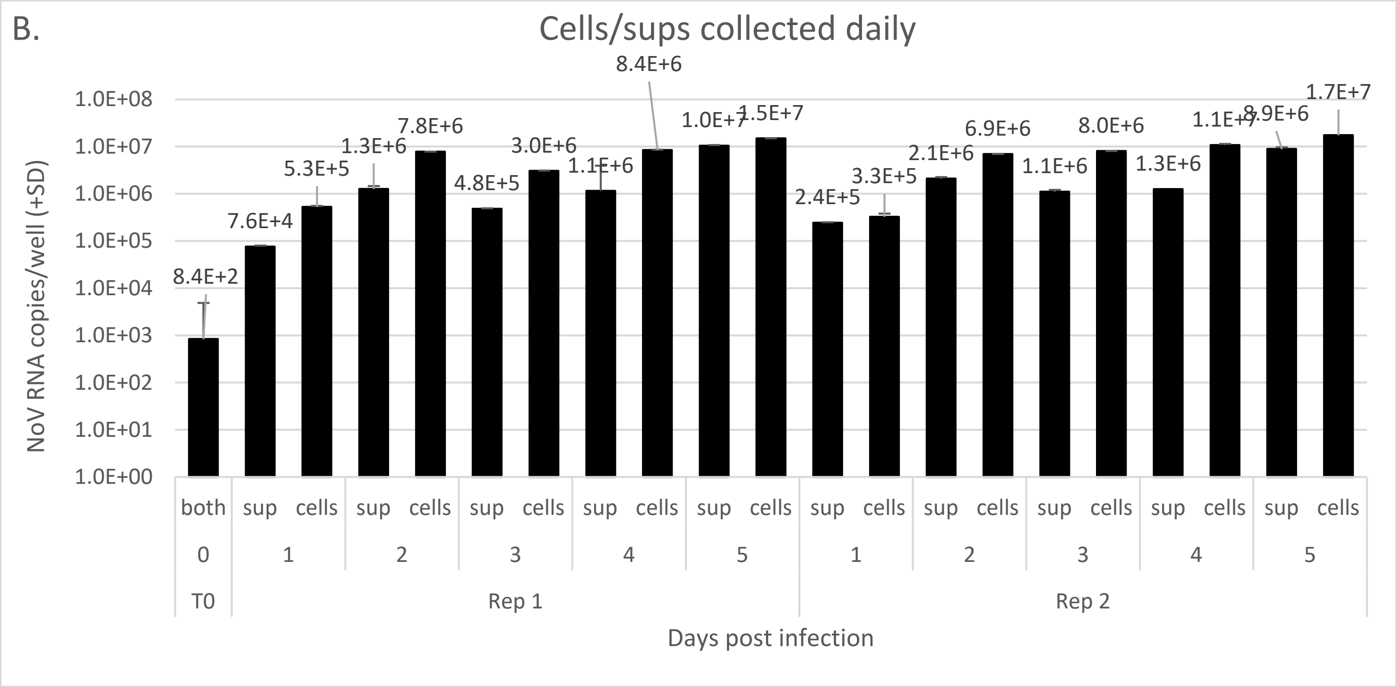

Figure S1. GII.4[P16] stool filtrate replication in cells and supernatants. A. Duplicate wells were infected, supernatants were collected (and replenished) daily post-infection, viral RNA quantitated by RT-qPCR and results presented for each of the duplicate wells. B. Duplicate wells were infected, and both cells and companion supernatants were collected daily, and viral RNA in the cell and supernatant fractions quantitated by RT-qPCR. One well was infected and collected immediately after virus adsorption and washing (0 days pi).

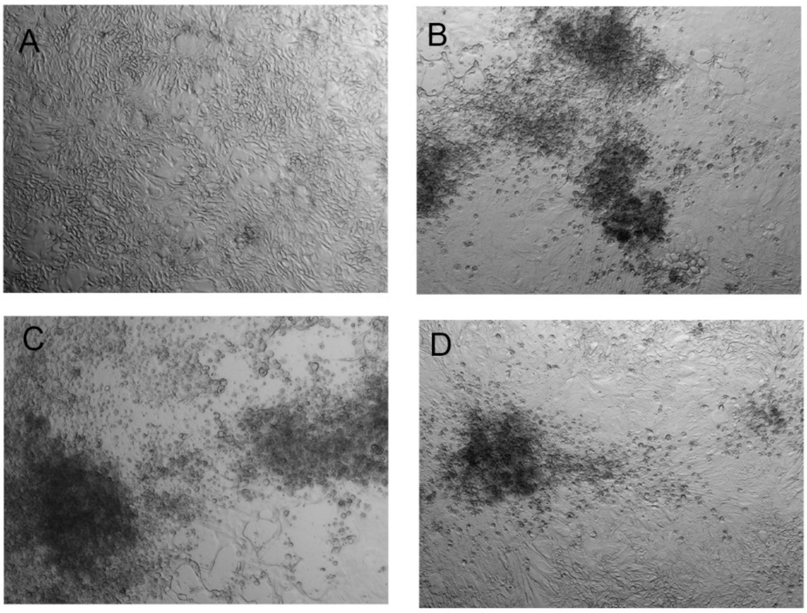

Figure S2. GII.4[P16] NoV-infected cells sometimes cause cytopathic effect in J2 HIEs. A. Mock-infected J2 HIE monolayers under brightfield imaging. B-D. GII.4[P16] NoV-infected J2 HIE monolayers 3 dpi under brightfield imaging at 5x magnification. Note the intact monolayer underneath discrete areas of dark/dense cells that have become unattached from the monolayer.

For passaging studies, an initial dilution of 1:200 of the GII.4[P16] strain was used to infect a total of 30 wells, which were separated into 3 replicates of 8 wells each, plus 2 wells to quantify the 0 h timepoint for each replicate. Additional media was added on day 2 post infection, as in the $TCID_{50}$ assay, and plates were frozen at 5 dpi after observing if cpe was present. After freezing and thawing 3x, cpe-positive and negative wells were pooled for each replicate, sonicated briefly, and microcentrifuged to remove cell debris. If no cpe was observed for a given passage, wells were pooled according to cpe positivity or negativity determined on the previous passage. A minimum of 50 µl was used for RNA extraction, and a 1:10 dilution of cpe-positive or -negative virus from each replicate was used to infect another set of monolayers, regardless of what the viral copy number was. For both the $TCID_{50}$ and the passaging studies, a minimum of 4 wells was mock-infected and included on the 5 dpi plate for comparison of cell morphology between infected and uninfected wells and were confirmed by qRT-PCR to be NoV-negative.

Passage of GII.4[P16] strain

During our initial investigations we observed robust replication of the GII.4[P16] NoV that also included, albeit inconsistently, the induction of a cytopathic effect (see Figure S2). We wanted to see if this virus would be amenable to longer passaging than has been previously reported in the J2 line (4 passages; {Ettayebi, 2016 #181}). Our approach was to have three replicates in which we pooled/passaged virus from wells that exhibited a cytopathic effect versus those that did not exhibit this effect, toward the goal of selecting for a cell culture-adapted mutant with a cpe+ phenotype (Fig. S2). Each subsequent passage was infected with a 1:10 dilution of the previous passage's pooled, sonicated, and clarified (by microcentrifugation to remove cellular debris). While the cytopathic effect was observed in all wells at passage 1 and passage 2, by passage 4 only a fraction of wells retained a cytopathic effect in all of the replicates, and by passage 5 no cytopathic effect was observed in any wells of any replicates. As shown in Fig. S2B, we were able to increase the number of passages that produced infectious virus from previous studies to 5-7 passages depending on the replicate, but by passage 8 there was no detectable virus in any of the three replicates.

A

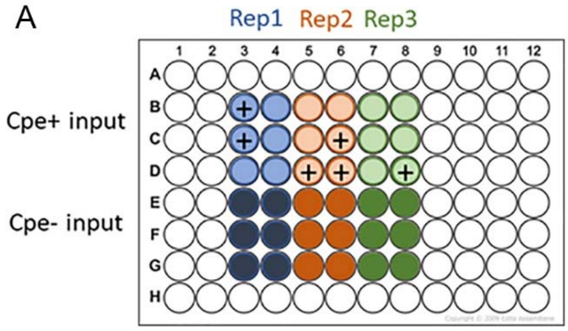

B.	Fold-increase of virus (5 dpi/0 hpi)		
	Rep 1	Rep 2	Rep 3
p1+	21885	17756	39003
p2+	61	63	415
p3+	232	1586	1209
p3-	22	0	1
p4+	9321	254	70
p4-	414	735	100
p5+	5438	3	19
p5-	49	1	0
p6+	6135	87	0
p6-	19	0	0
p7+	227	0	0
p7-	14	0	0
p8+	0	0	0
p8-	0	0	0

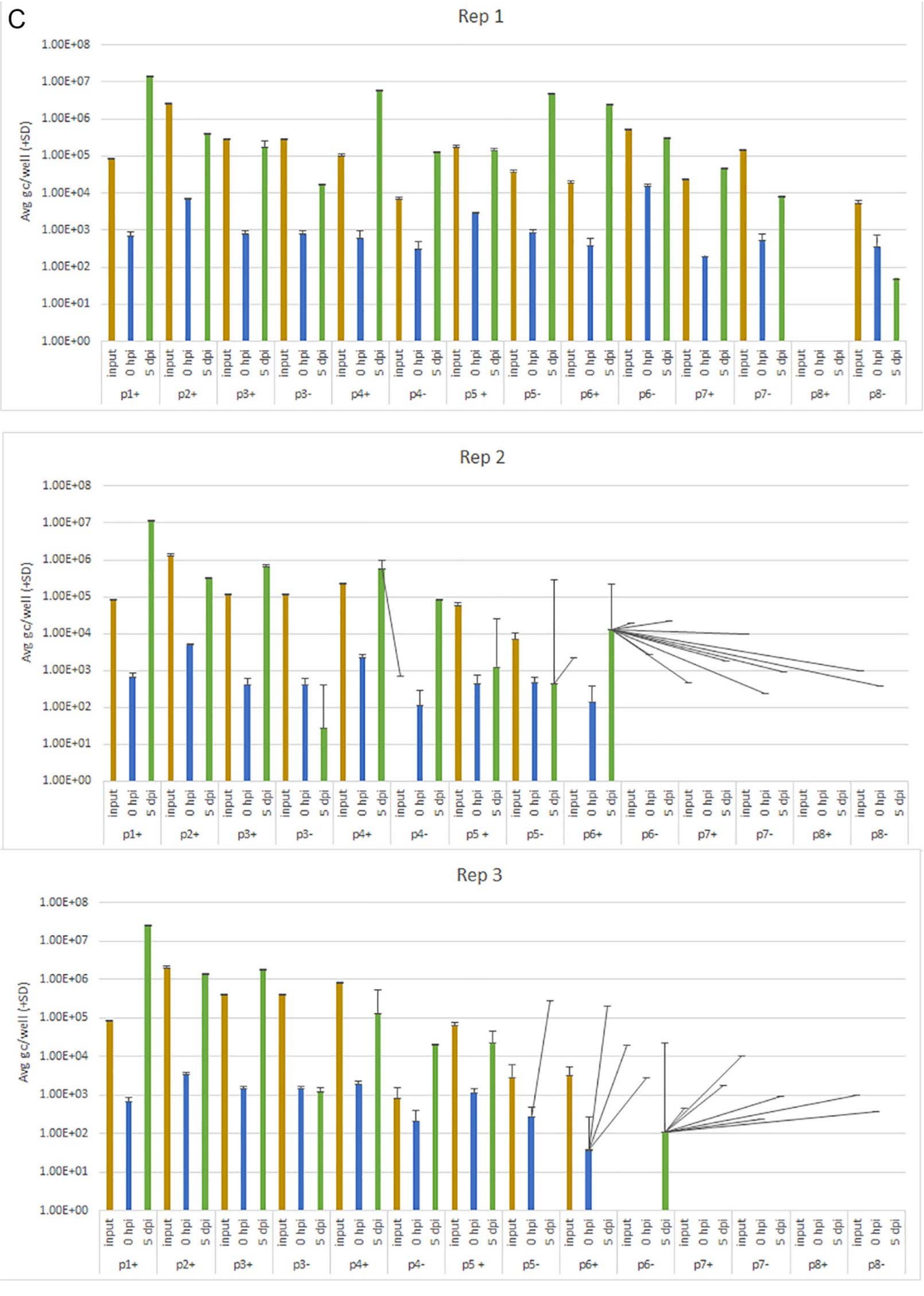
C
Rep 1
Rep 2
Rep 3
Avg gc/well (+SD)
1.00E+00
1.00E+01
1.00E+02
1.00E+03
1.00E+04
1.00E+05
1.00E+06
1.00E+07
1.00E+08
input
0 hpi
5 dpi
p1+
p2+
p3+
p3-
p4+
p4-
p5 +
p5-
p6+
p6-
p7+
p7-
p8+
p8-

Figure S3. Passage of GII.4[P16] virus in J2 HIEs. A. Illustration of a 96-well plate scheme used for virus infection/virus passage for production of passage 4 virus. In this example, a 1:10 dilution of passage 3 cpe+ and cpe- viruses were used to infect 6 wells of each replicate as indicated. After 5 days of infection, 2 wells from Rep1 were pooled for cpe+ virus (+), and the remaining 10 were pooled for cpe- virus. Three cpe+ wells were pooled from Rep2 and only one cpe+ well was kept for Rep3 for cpe+ viruses. No cpe was observed at ≥ passage 5 for any of the replicates; wells were then pooled based on cpe status of previous passages. B. Fold-increase of virus passages in 3 replicates. 2/3 reps showed successful infection beyond passage 4. Only Rep1 showed virus present at passage 7, which was undetectable by passage 8. C. Average input (orange bars), 0 hpi (blue bars) and 5 dpi (green bars) genome copies/well for each replicate +SD.

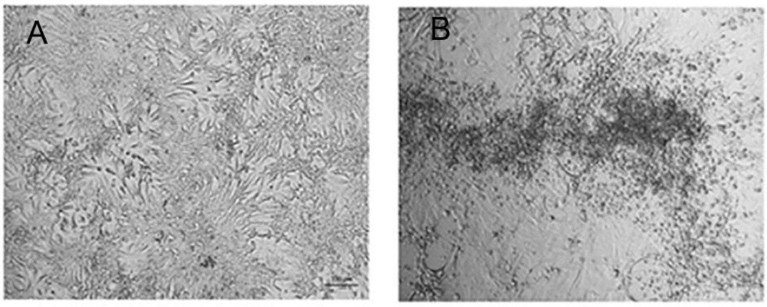

Figure S4. Cytopathic effect in J2 enteroids infected with GII.4[P16], but not GII.6[P7], norovirus isolated from frozen raspberries. A: GII.6[P7], recovered from spiked berry extract, shown at 3 dpi, 5x magnification. B: GII.4[P16], recovered from spiked berry extract, shown at 3 dpi, 5x magnification.
